# Virtual planning in orthognathic surgery among Swedish maxillofacial surgeons: a retrospective register-based cohort study

**DOI:** 10.1007/s10006-026-01524-w

**Published:** 2026-02-20

**Authors:** Maria Spändbåge, Helena Stureson, Esmeralda Bäckström, Mats Sjöström

**Affiliations:** 1 Department of Odontology, Umeå University, Umeå, Sweden; 2 Department of Odontology, Oral and Maxillofacial Surgery, Umeå University, Umeå, Sweden

**Keywords:** Orthognathic surgery, Virtual surgical planning, Conventional surgical planning, Three-dimensional surgical planning, Two-dimensional surgical planning

## Abstract

**Purpose:**

The integration of virtual surgical planning (VSP) into orthognathic surgery has improved preoperative visualization, surgical precision, and patient-specific outcomes. However, limited information is available regarding the utilization of VSP in Swedish maxillofacial clinics. This study primarily aimed to provide statistical analysis and descriptive statistics on orthognathic surgical parameters in Sweden between 2017 and 2024 and focused on comparing utilization between VSP and conventional surgical planning (CSP).

**Methods:**

In this retrospective register-based cohort study, statistical analyses were carried out using anonymized data from 2 386 non-syndrome associated patients registered in the Swedish National Registry for Orthognathic Surgery during the years 2017 to 2024.

**Results:**

The use of VSP among Swedish oral and maxillofacial surgeons significantly increased during the study period. A significant difference was found (*p* < 0.001) between VSP and CSP regarding surgery type, intraoperative blood loss for bimaxillary surgery, and surgery time for bimaxillary surgery. VSP is predominantly used in bimaxillary surgery, whereas CSP is more commonly used in single-jaw surgery.

**Conclusion:**

The findings of this study demonstrate a significant increase in the adoption of VSP among Swedish maxillofacial surgical units between 2017 and 2024, particularly in planning bimaxillary surgeries compared to single-jaw surgeries. In addition, VSP may reduce intraoperative bleeding and shorten surgery time in bimaxillary surgeries compared with CSP.

## Introduction

Dentofacial deformities are abnormal proportions of the maxillomandibular complex that result in a strongly unfavorable occlusal relationship [1]. Such deformities lead to varying impairments of jaw function, with associated pain and temporomandibular disorders, negatively impacting masticatory function [2]. Nevertheless, psychological and aesthetic factors strongly influence the quality of life of patients with dentofacial deformities [3, 4].

Orthognathic surgery aims to establish favorable maxillofacial function, normalize facial harmony, and increase health-related quality of life [4–6]. The various indications for surgery include craniofacial deformities, such as cleft lip and palate, increased or reversed overjet, open bite, complete scissors bite, sleep apnea, and skeletal anomalies [7].

Before surgical correction, it is crucial to conduct a thorough analysis and develop a detailed plan for the surgical procedure to be performed using conventional surgical planning (CSP) or virtual surgical planning (VSP). CSP is performed with two-dimensional radiographs for cephalometric evaluation, plaster dental cast evaluation, and splint preparation [1]. The method was designed before digitalization and is still widely used today. In contrast, VSP enables surgeons to create a virtual anatomical model of the patient’s head and neck, incorporating facial soft tissue, underlying bone structures, and occlusion. A computed tomography (CT) or cone beam computed tomography (CBCT) image is imported into computer software to create a virtual three-dimensional (3D) model for planning [8]. The 3D model can also visualize potential postoperative outcomes and facilitate determining treatment options for the patient. Individualized wafers are manufactured using computer-aided design and computer-aided manufacturing [9]. The interocclusal relationship is obtained by using either a 3D surface scanner on traditional cast models of the patient’s teeth or an intraoral scanner directly on the patient’s teeth [10].

Depending on the desired outcome, different surgical techniques are used on both the maxilla and mandible, often foregoing orthodontic treatment and, not uncommonly, are some orthodontics continuing after surgery for final adjustments [11]. In Sweden, Le Fort I osteotomy is the most common maxillary osteotomy and the bilateral sagittal split osteotomy in the mandible [12, 13]. In cases of extensive deviations between the dental arches, osteotomies are performed on both the maxilla and mandible, referred to as bimaxillary surgery.

The average prevalence rate of orthognathic procedures between 2010 and 2014 was reported to be 6.3 per 100 000 persons, with 39.4% of patients undergoing bimaxillary surgery, and the mean age at the time of surgery was 22 years [13]. The first Swedish quality registry for orthognathic surgery (NROK) started in 2017, achieving 91% coverage and good adherence [12]. Of the 25 maxillofacial surgery units in Sweden that perform orthognathic surgery [13], 18 are affiliated with NROK. The NROK receives approximately 900 to 1 000 new registrations annually.

Orthognathic surgery is generally considered a safe procedure with a very low incidence of complications. However, both intraoperative and postoperative complications can occur. These complications, including surgical blood loss, neurosensory deficits of the inferior alveolar nerve, temporomandibular joint issues, relapse, and local irritation or infection related to osteosynthesis materials, can range from mild to severe [14].

The recent literature presents conflicting evidence regarding whether VSP or CSP is more cost-effective and less time-consuming. One study indicated that VSP is significantly more cost-effective than CSP in bimaxillary surgery considering reduced preoperative laboratory work, shorter surgery time, and lower planning costs [15]. Another highlighted the disadvantages of VSP, including the cost of CBCT scans and software, as well as longer planning time [16]. The average planning time for CSP was reported to be 20 min per patient, whereas VSP required 38 min on average [16]. However, over the 1-year evaluation period, planning time with VSP decreased as experience increased, highlighting a learning curve [16].

The techniques demonstrate comparable accuracy in predicting hard-tissue outcomes, but VSP exhibits superior accuracy in predicting soft-tissue outcomes [16]. Though both methods have been reported to effectively achieve sagittal correction and high patient satisfaction with results, patients operated on with VSP had better midline alignment, less occlusal plane misalignment, and more balanced ramus inclination, and a greater proportion of patients reported being “very satisfied” with the outcomes [17]. Surgeons also reported achieving more desirable results with VSP, particularly in terms of mandibular contour symmetry [18].

Furthermore, studies have shown that VSP provides greater accuracy in predicting anterior maxillary outcomes and offers advantages when planning for correction of facial asymmetry despite both CSP and VSP demonstrating strong overall predictive capabilities for facial outcomes [19]– [20]. Notably, improvements in facial aesthetics have been observed in patients regardless of the planning method [21].

Both CSP and VSP are currently used for planning orthognathic surgery, but knowledge of their actual use in Sweden remains limited. Despite studies suggesting potential advantages of VSP in surgical precision, patient satisfaction, and cost-effectiveness, its adoption across surgical units and temporal trends remain largely unexplored. By analyzing these patterns, we aim to contribute to clinical decision-making, resource use, and favorable patient outcomes, as well as increasing understanding the impact of digitalization in surgical planning on efficiency and accuracy, thereby improving patient care for those with dentofacial deformities.

This retrospective register-based cohort study aimed to provide a descriptive statistical analysis of orthognathic surgeries in Sweden between 2017 and 2024. The primary objective was to evaluate the use of CSP and VSP among Swedish oral and maxillofacial surgical units that perform orthognathic surgery and to investigate potential temporal trends in their use. The hypothesis was that VSP is more frequently used during bimaxillary surgery. The study also aimed to evaluate the number of included patients, sex distribution, mean surgery time, intraoperative blood loss, and mean age at the time of surgery, as well as provide a descriptive analysis of the numbers of single-jaw and bimaxillary surgeries.

## Materials & methods

### Study population and data selection

**Fig. 1:**
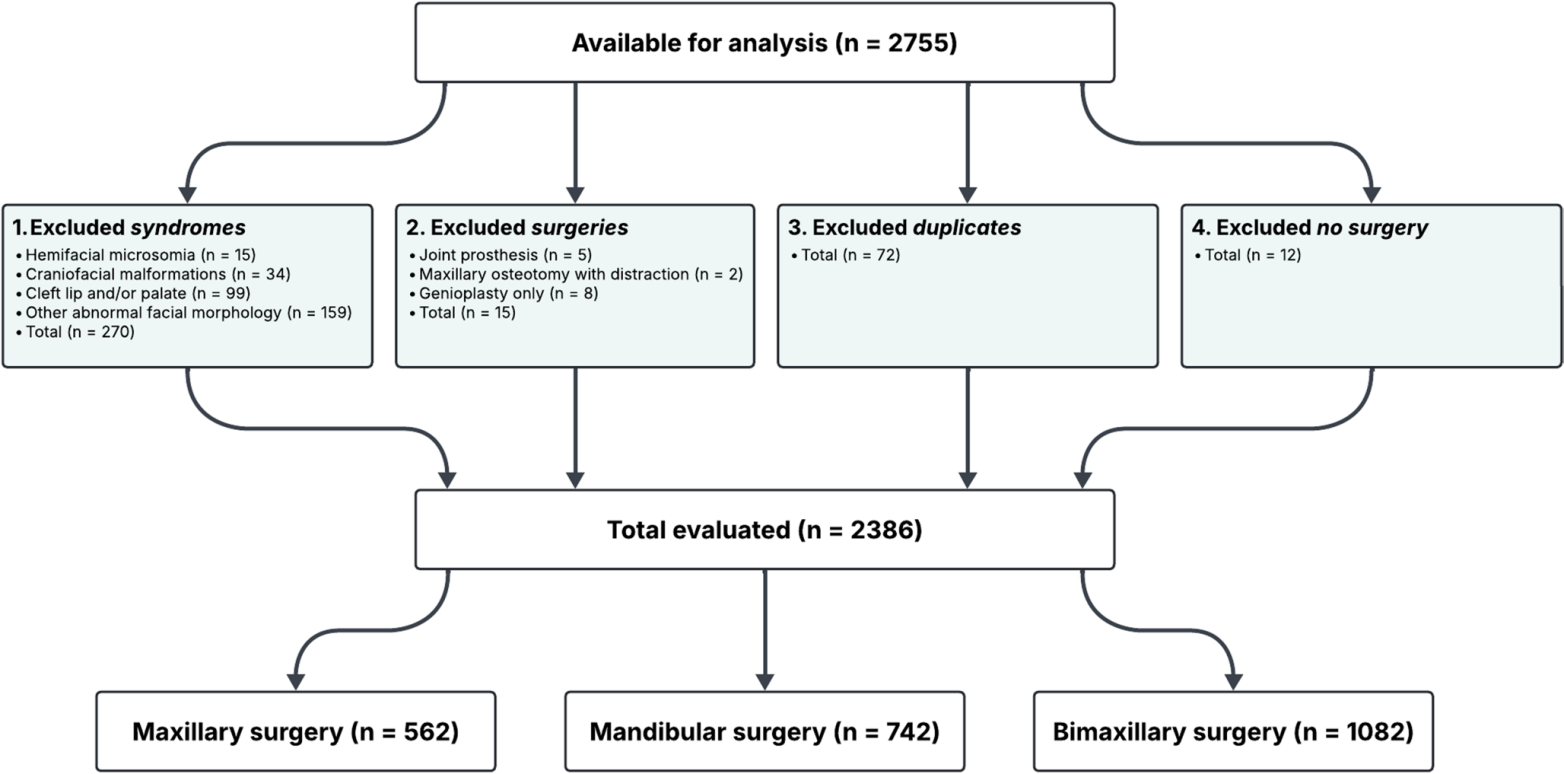
Flowchart of data selection and exclusion

In October 2024, NROK provided an anonymized dataset consisting of 2 755 sequence numbers registered from 2017 to 2024. Each unique sequence number corresponds to a patient and serves as an anonymized identifier to evaluate individual patient data without compromising patient confidentiality. The sequence numbers were reviewed for inclusion according to predefined criteria (Fig. 1).

First, entries associated with craniofacial syndromes or developmental anomalies were removed. A total of 307 diagnostic labels meeting the exclusion criteria were identified; 37 patients had more than one diagnosis recorded and were counted only once. Thus, this categorization represented 270 unique patients who were excluded from the study population.

Next, surgery types linked to excluded diagnoses, patients who underwent only genioplasty, and duplicate registrations were excluded. Duplicate entries were identified as the same sequence number appearing in multiple rows in the dataset, indicating that some patients had undergone orthognathic surgery on more than one occasion during the study period. Only the first recorded surgery per patient was included to ensure that each patient contributed only one observation to the analysis and to avoid overrepresentation of individuals with repeated procedures. In addition, 12 patients had no recorded surgery type data, possibly due to our exclusion criteria for the NROK dataset. These patients were not included in the statistical analysis.

After applying all exclusion criteria and removing duplicates and entries missing surgery type, 2 386 sequence numbers remained for analysis. These were categorized (see Appendix A) into maxillary surgery (*n* = 562), mandibular surgery (*n* = 742), and bimaxillary surgery (*n* = 1 082).

### Statistical analysis

Statistical analyses were performed using IBM SPSS Statistics (version 28.0, IBM Corp, Armonk, NY, USA). Cross-tabulation was used to analyze potential patterns and correlations for CSP and VSP, as well as the variables used in this study. The significance level was set at *p* < 0.05.

A Pearson chi-squared test was used to assess the significance of differences between CSP and VSP for the variables of interest. The variables of interest were the number of surgeries, number of surgery types (maxillary, mandibular, and bimaxillary), patient sex, intraoperative blood loss (divided into single-jaw and bimaxillary surgery groups; <200 ml, 200–500 ml, > 500 ml), and surgery time (divided into single-jaw and bimaxillary surgery groups; <2 h, 2–4 h, 4–6 h, > 6 h).

Sixty-seven patients with no intraoperative blood loss data recorded and 32 patients with no surgery time data recorded were excluded from the statistical analyses of those variables.

## Results

### Descriptive statistics

The final sample consisted of 2 386 patients, including 1 109 (46.5%) males and 1 277 (53.5%) females ranging in age from 15 to 71 years, with a mean age of 24.05 years at the time of surgery. Of these 2 386 patients, 1 440 (60.4%) underwent surgery with CSP and 946 (39.6%) with VSP. The descriptive statistics and analysis are provided in Table 1.

**Table 1:** Descriptive statistics and analysis of the included data

Variable of interest	CSP, *n* (%)	VSP, *n* (%)	Total, *n* (%)	*P*-value ^a^
Surgery type				< 0.001*
Maxillary	448 (31.1)	114 (12.1)	562 (23.6)	
Mandibular	594 (41.3)	148 (15.6)	742 (31.1)	
Bimaxillary	398 (27.6)	684 (72.3)	1 082 (45.3)	
Total	1 440 (100)	946 (100)	2 386 (100)	
Sex				0.199
Male	654 (45.4)	455 (48.1)	1 109 (46.5)	
Female	786 (54.6)	491 (51.9)	1 277 (53.5)	
Total	1 450 (100)	946 (100)	2 386 (100)	
Blood loss, single-jaw surgery				0.233
< 200 ml	663 (65.9)	181 (69.6)	844 (66.7)	
200–500 ml	329 (32.7)	73 (28.1)	402 (31.8)	
> 500 ml	14 (1.4)	6 (2.3)	20 (1.6)	
Total	1 006 (100)	260 (100)	1 266 (100)	
Blood loss, bimaxillary surgery				< 0.001*
< 200 ml	70 (18.5)	203 (30.1)	273 (25.9)	
200–500 ml	242 (63.9)	409 (60.7)	651 (61.8)	
> 500 ml	67 (17.7)	62 (9.2)	129 (12.3)	
Total	379 (100)	674 (100)	1 053 (100)	
Surgery time, single-jaw				0.124
< 2 h	553 (53.9)	129 (49.6)	682 (53.0)	
2–4 h	462 (45.0)	125 (48.1)	587 (45.6)	
4–6 h	10 (1.0)	4 (1.5)	14 (1.1)	
> 6 h	1 (0.1)	2 (0.8)	3 (0.2)	
Total	1 026 (100)	260 (100)	1 286 (100)	
Surgery time, bimaxillary				< 0.001*
< 2 h	13 (3.4)	24 (3.5)	37 (3.5)	
2–4 h	248 (64.1)	510 (74.9)	758 (71.0)	
4–6 h	107 (27.6)	135 (19.8)	242 (22.7)	
> 6 h	19 (4.9)	12 (1.8)	31 (2.9)	
Total	387 (100)	681 (100)	1 068 (100)	
*CSP*, conventional surgical planning; *VSP*, virtual surgical planning. *p < 0.05. ^a^ Pearson’s chi-squared.				

### Surgery type

The analysis revealed a significant difference between the type of surgery performed and the planning method (*p* < 0.001). VSP was predominantly used for bimaxillary surgery (72.3%), and CSP was primarily used for single-jaw surgery, both maxillary (31.1%) and mandibular (41.3%).

### Sex

A comparison between sex and the utilization of VSP and CSP showed no significant difference (*p* = 0.199). Male patients accounted for 45.4% and female patients for 54.6% of the CSP group. In the VSP group, the sex distribution was 48.1% males and 51.9% females.

### Bleeding

Distribution analysis of intraoperative blood loss during single-jaw surgeries showed no significant difference between CSP and VSP (*p* = 0.223). However, a significant difference was observed between CSP and VSP in the bimaxillary surgery groups (*p* < 0.001). For intraoperative blood loss, the CSP group showed that 18.5% of patients lost < 200 ml, 63.9% lost 200–500 ml, and 17.7% lost > 500 ml. In comparison, the VSP group demonstrated 30.1% with < 200 ml blood loss, 60.7% with 200–500 ml, and 9.2% with > 500 ml. Thus, the CSP group had a higher proportion of patients with major blood loss (> 500 ml), whereas the VSP group had a larger proportion with low blood loss (< 200 ml).

### Surgery time

Single-jaw surgeries were generally completed in under 2 h, while most bimaxillary procedures required 2–4 h. Surgery time for single-jaw procedures was not significantly different between CSP and VSP (*p* = 0.124). In contrast, bimaxillary surgery time showed a significant difference between the groups (*p* < 0.001). In the CSP group, 3.4% of surgeries lasted < 2 h, 64.1% lasted 2–4 h, 27.6% lasted 4–6 h, and 4.9% exceeded 6 h. In the VSP group, 3.5% of surgeries lasted < 2 h, 74.9% lasted 2–4 h, 19.8% lasted 4–6 h, and 1.8% exceeded 6 h. Bimaxillary surgeries planned with VSP demonstrated fewer procedures exceeding 6 h and a higher proportion completed within 2–4 h compared to CSP.

### Temporal trends of utilization of VSP and CSP

**Fig. 2:**
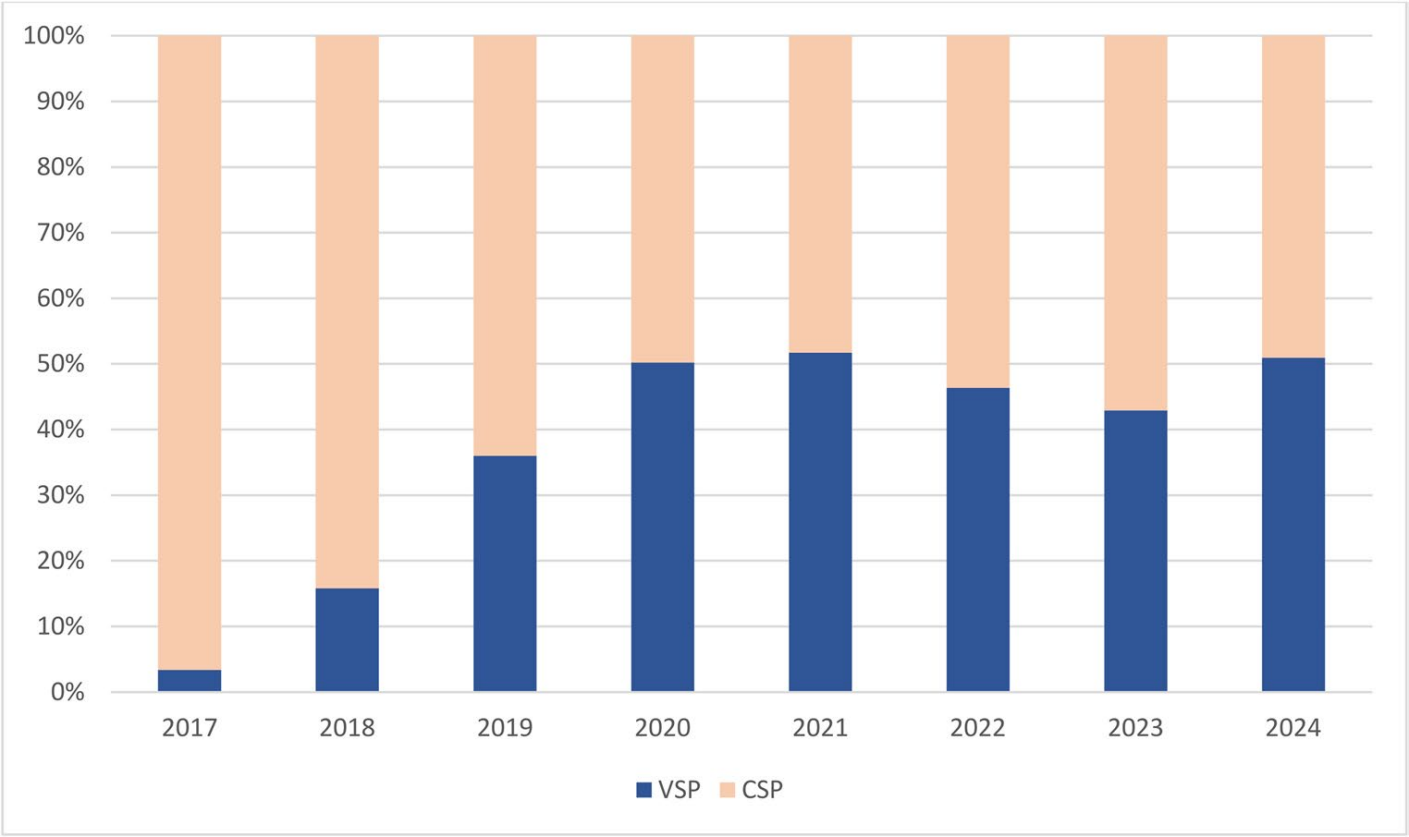
Relative utilization of conventional surgical planning (CSP) and virtual surgical planning (VSP) from 2017 to 2024

**Fig. 3:**
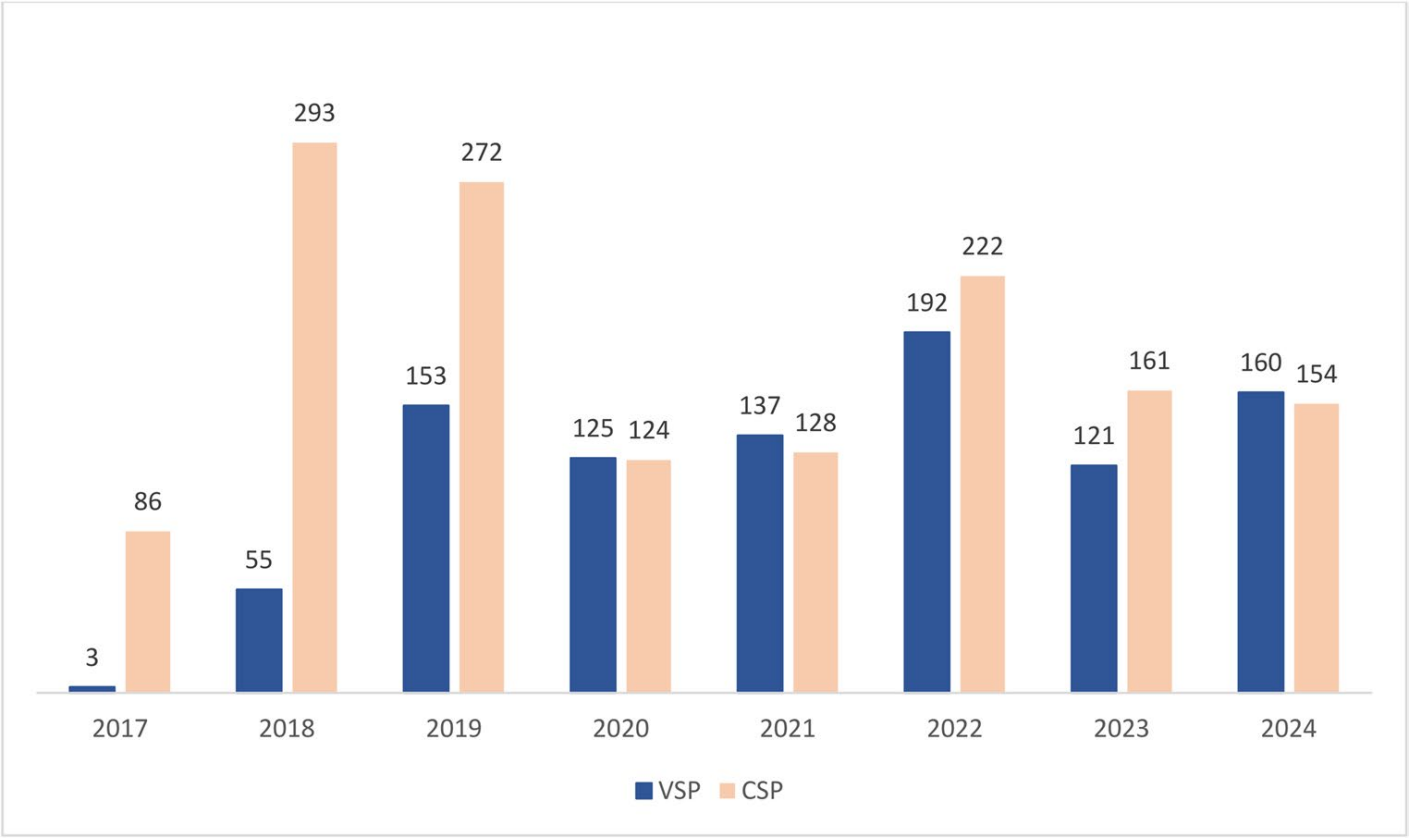
Number of surgeries performed with conventional surgical planning (CSP) and virtual surgical planning (VSP) annually from 2017 to 2024

We observed a significant increase in the utilization of VSP between 2017 and 2024 compared to CSP (*p* < 0.001; Figs. 2 and 3). From 2017 to 2020, VSP use increased by 46.8% points, from 3.4% to 50.2%, with an equivalent decrease in CSP use from 96.6% to 49.8%. From 2020 to 2024, the difference between planning methods plateaued.

## Discussion

The purpose of this register-based retrospective study was to compare VSP and CSP in orthognathic surgeries performed in Sweden between 2017 and 2024, focusing on general surgical parameters and usage trends. To the best of our knowledge, this study is the first to compare the utilization of VSP and CSP in orthognathic surgeries using systematically registered data from NROK.

### Bimaxillary vs. single-jaw surgery

This study demonstrates a significant association between planning method and surgery type, with VSP use being substantially more prevalent in bimaxillary surgeries. A likely explanation is that bimaxillary surgery generally involves greater technical complexity and, therefore, benefits from enhanced accuracy, visualization, and predictability provided by VSP. Another explanation can be attributed to the individual clinic’s approach to the planning system. Within the maxillofacial surgical care panorama, virtual technology is currently used in implant treatment, orthognathic surgery, trauma treatment, and temporomandibular joint reconstruction. In some clinics, virtual planning is performed collaboratively by surgeons and biomedical engineers, which may streamline the process while improving precision and reproducibility. A further hypothesis is that a case may initially be planned as a single-jaw procedure. However, during VSP, minor asymmetries may become apparent. Consequently, the surgeon may decide to perform bimaxillary surgery to address these findings, despite the original plan involving only one jaw. VSP may therefore contribute to an increased rate of bimaxillary surgeries. In contrast, with CSP, such minor asymmetries may not be detected, and the surgeon may consequently adhere to the original single-jaw surgical plan.

The continued use of CSP in single-jaw surgery may be explained by variations in cost and resource availability among clinics. In some settings, VSP may be more expensive when the planning process is outsourced to external providers rather than performed in-house. Consequently, the choice of planning method may depend on each institution’s financial and logistical circumstances, with clinics often reserving VSP for more demanding cases. There is varying evidence on the cost-efficiency of VSP [15, 16]. Results from our study indicate that VSP in bimaxillary surgery leads to shorter surgery time. However, further research is needed to investigate the associated costs for Swedish surgery units and to determine the extent to which VSP is cost-benefit efficient compared with CSP.

Both planning time and surgery time likely depend on the surgeon’s experience and established workflow. Further studies are needed to estimate whether VSP or CSP is less time-consuming. Overall, this study’s findings suggest that VSP utilization by surgery type is influenced by surgical complexity, which may have a bidirectional relationship. However, other factors, such as economic, logistical, and time-related considerations, may vary across clinical environments and influence decision-making.

### Sex

The results indicate that sex does not influence the choice of planning method. Men and women had comparable proportions of virtual and conventional planning, and the difference was not significant (*p* = 0.199). This suggests that the adoption of VSP is likely driven by other factors.

### Intraoperative blood loss

Intraoperative blood loss varied between CSP and VSP during bimaxillary surgeries but not for single-jaw surgeries. Greater VSP use was associated with lower blood loss (200 ml) compared with CSP, which was more prevalently used in surgeries with blood loss ≥ 200 ml. A possible explanation is the precision of VSP and shorter surgery. Another factor to speculate on, is whether the bimaxillary surgery procedure itself could have impact on the surgeon’s decision to prescribe anticoagulants. Some clinics routinely administer anticoagulants during surgery, whereas others do not. Further investigation is needed to evaluate the relationship between planning method and intraoperative blood loss.

### Surgery time

In bimaxillary surgeries VSP use was associated with a shorter surgery duration compared with CSP (Table 1). VSP may provide the surgeon with better preoperative visualization of skeletal movement, enhance splint accuracy, and reduce intraoperative adjustments, thereby improving workflow and reducing surgical inefficiency. A longer surgery time with CSP indicates that the method may lead to lower intraoperative efficiency. Minor inaccuracies may facilitate error propagation, less preoperative visualization, and greater intraoperative modifications, which cumulatively lead to extended surgical duration. Shorter surgery time is clinically desired because of the general association with lower complication rates, including excessive intraoperative blood loss.

### Trends in the utilization of virtual surgical planning

Several factors may explain the significant increase in VSP utilization observed during the study period. The introduction of VSP computer systems to Swedish maxillofacial surgeons in 2017 likely attracted early adopters and stimulated initial growth. Implementing VSP at a surgical clinic requires access to intraoral scanners, specialized computers, a CBCT or CT machine, and support from an IT department connected to the clinic. These requirements entail additional financial investments, which may limit adoption at some clinics. However, further studies are needed to assess the actual cost-effectiveness of VSP. Moreover, individual surgeons’ preferences, experience, and interest in digital planning technologies may influence the extent of VSP use. Collectively, these factors may account for the subsequent plateau in VSP adoption.

We observed a substantial decline in orthognathic surgeries performed with both CSP and VSP in 2020 and 2021, which correlates with the COVID-19 pandemic and indicates a significant impact on elective surgical procedures during this period (Fig. 3). The pandemic may also have contributed to the subsequent stagnation in VSP utilization. As clinics prioritized catching up on postponed surgeries and restoring surgical volumes, the introduction of new workflows, such as VSP, may have been deprioritized. Adopting a new planning system requires time for training and adaptation, and a temporary reduction in productivity is often inevitable during the learning phase. Therefore, these organizational and practical factors could have further delayed the continued expansion of VSP after the pandemic.

### Limitations

The risk of confounding and selection bias should be considered in a retrospective cohort study without randomization. Given that participants were not randomly allocated to the surgical planning methods, there may be systematic differences between them that could have influenced the results. The potential confounding due to differences in surgery type groups was mitigated by dividing surgery time and intraoperative blood loss into single-jaw surgery and bimaxillary surgery groups. A surgeon’s choice of planning method may potentially be influenced by preferences for a more traditional or modern approach, confidence in handling complex cases with one method or another, access, or familiarity. This can potentially lead to performance bias if a surgeon, for some reason, chooses only one method. Another surgeon-related confounding factor might be underreporting surgeries to NROK, as they are not obligated to do so. Although we cannot eliminate underreporting, the large data sample reduces its possible impact.

### Future research

Clinical decision-making and adoption could be studied through a national survey, which could provide valuable insights into why different clinics choose to adopt or abstain from using VSP. This study did not include a cost comparison between VSP and CSP, but it is still important to consider not only how well a method works clinically but whether it is cost-effective. Data from NROK may be used for further studies on the cost-effectiveness of the two planning methods.

As digital workflows continue to evolve, user-friendliness and automation are improving rapidly. The integration of artificial intelligence (AI) into surgical planning might further accelerate the process. Future studies could investigate how AI-assisted planning tools influence both efficiency and clinical outcomes.

## Conclusion

This study demonstrated a significant increase in the adoption of VSP among Swedish maxillofacial surgical units between 2017 and 2024, particularly in planning bimaxillary surgeries compared to single-jaw surgeries. In addition, VSP may reduce intraoperative bleeding and shorten surgery time in bimaxillary surgeries compared with the use of CSP.

## Data Availability

The datasets generated and analyzed during the current study are not publicly available under the Swedish Journal Act but are available from the corresponding author upon reasonable request.

## Appendix

### Surgery type groupings

#### Maxillary surgery

Le Fort I, Le Fort II, Le Fort III, and surgically assisted rapid maxillary expansion.

#### Mandibular surgery

Intraoral vertical ramus osteotomy, extraoral vertical ramus osteotomy, bilateral sagittal split ramus osteotomy, segmental osteotomy, and mandibular osteotomy

#### Bimaxillary surgery

Combined maxillary and mandibular surgery.
